# Temporally deuterogenic plasmonic vortices

**DOI:** 10.1515/nanoph-2023-0931

**Published:** 2024-02-21

**Authors:** Xinyao Yuan, Quan Xu, Yuanhao Lang, Zhibo Yao, Xiaohan Jiang, Yanfeng Li, Xueqian Zhang, Jiaguang Han, Weili Zhang

**Affiliations:** Center for Terahertz Waves, College of Precision Instrument and Optoelectronics Engineering, Key Laboratory of Optoelectronic Information Technology (Ministry of Education of China), Tianjin University, Tianjin 300072, China; Guangxi Key Laboratory of Optoelectronic Information Processing, School of Optoelectronic Engineering, Guilin University of Electronic Technology, Guilin 541004, China; School of Electrical and Computer Engineering, Oklahoma State University, Stillwater, OK 74078, USA

**Keywords:** deuterogenic plasmonic vortices, spatiotemporal dynamics, orbital angular momentum

## Abstract

Over the past decade, orbital angular momentum has garnered considerable interest in the field of plasmonics owing to the emergence of surface-confined vortices, known as plasmonic vortices. Significant progress has been made in the generation and manipulation of plasmonic vortices, which broadly unveil the natures of plasmonic spin–orbit coupling and provide accessible means for light–matter interactions. However, traditional characterizations in the frequency domain miss some detailed information on the plasmonic vortex evolution process. Herein, an exotic spin–orbit coupling phenomenon is demonstrated. More specifically, we theoretically investigated and experimentally verified a temporally deuterogenic vortex mode, which can be observed only in the time domain and interferes destructively in the intensity field. The spatiotemporal evolution of this concomitant vortex can be tailored with different designs and incident beams. This work extends the fundamental understanding of plasmonic spin–orbit coupling and provides a unique optical force manipulation strategy, which may fuel plasmonic research and applications in the near future.

## 1 Introduction

Vortex beams carrying orbital angular momentum [1], [2], [3], [4] (OAM) have attracted continuous interest due to their unique helical phase front characterized by the Hilbert factor e^i*lφ*^, where *l* (an arbitrary integer) and *φ* are the topological charge and azimuthal angle, respectively. Notably, plasmonic vortices have also undergone substantial development owing to their potential for on-chip particle manipulation and information processing [5], [6], [7], [8]. Typically, these plasmonic vortices refer to surface plasmons [9], [10], [11], [12] (SPs) characterized by a dark spot and phase singularity at the center, while exhibiting strong confinement of the OAM in the evanescent field region with subwavelength dimensions. Thus, understanding and controlling such plasmonic vortices will pave the way for prospective applications, such as light–matter interactions [13], quantum communication [14], [15], super-resolution imaging [16], and plasmonic spanners for rotating both dielectric and metallic particles [17], [18], [19].

With the advances in sub-wavelength optics and fabrication technology, studies in metasurfaces have greatly facilitated the on-chip generation and manipulation of OAM [10], [20], [21], [22], [23], [24], [25], [26], [27]. Through constructing subwavelength artificial structures, along with the important spin–orbit coupling [28], [29], [30], the spin angular momentum (SAM) carried by an incident beam is converted to the plasmonic OAM and excites the desired plasmonic vortex. In addition, the spin–orbit interaction also inspires new mechanisms for flexible control of plasmonic vortices, such as spatiotemporal vortices with transverse OAM [31], [32], OAM multiplication in plasmonic vortex cavities [33] and spatiotemporal dynamic manipulation of plasmonic vortices [34]. Using a set of Archimedean spiral slits, researchers also reported the existence of a deuterogenic plasmonic vortex mode in the frequency domain [35]. These works provide a fundamental understanding of the near-field spin–orbit coupling, which promises to flexibly manipulate plasmonic vortices for emerging optical vortex-based technologies and further on-chip applications [36].

In this paper, we theoretically demonstrate and experimentally observe the existence of an unusual vortex mode in terahertz (THz) regime, which appears concomitantly with the conventional one only in the time domain and interferes destructively in the field intensity pattern. The detailed spatiotemporal dynamics of the plasmonic vortices is studied with the generalized Huygens–Fresnel principle in numerical simulations and near-field scanning THz microscopy (NSTM) in experiments. Thus, we can reveal the general process of spin–orbit coupling in plasmonic vortex lenses (PVLs). Meanwhile, flexible manipulation of the temporally deuterogenic vortex mode can be achieved through different designs. These unique spatial and temporal distributions of optical forces are expected to render new effects and functions when they are exploited in light–matter interactions.

## 2 Results

### 2.1 Temporally deuterogenic plasmonic vortices

PVLs consisting of rectangular slit-pair resonators milled in a metal film on a dielectric substrate are commonly used when designing metasurfaces for plasmonic vortex generation [23], [24], [37]. Generally, for every single pair in the design, two slit resonators are separated by a distance of *λ*_SP_/2 and arranged perpendicular to each other. All the slits have the same geometric dimensions but different orientation angles, and the rotations of the slits are defined as

$$\theta_{1,m}=g\varphi_{m}/2+\pi /2+\alpha_{0}$$

$$\theta_{2,m}=g\varphi_{m}/2+\alpha_{0}$$

where *θ*_1, *m*_ and *θ*_2, *m*_ represent the orientation angles of the inner and outer slit resonators of the *m*th slit-pair, respectively; *g* is a geometric phase factor describing the rotation order across a whole turn along the circle in the anticlockwise direction; *φ*_*m*_ is the azimuthal angle of the *m*th slit-pair with respect to the *x*-axis; and *α*_0_ is the initial orientation angle of the outer slit resonator at azimuthal *φ*_*m*_ = 0 (see Section 1, Supplementary Materials). When circularly polarized (CP) light illuminates the sample from the substrate side, since each slit can launch SPs with a different initial spin-dependent phase depending on its orientation angle *θ*, a phase shift equal to 2*gσπ* is introduced, known as the Pancheratnam–Berry phase or geometric phase [24], [38], [39], [40], [41], where $\sigma \in \left\{+,-\right\}$ stands for the spin-direction of the right- and left-handed CP (RCP and LCP) light. Another part of phase shift caused by the fixed change of the azimuthal angle −2*σπ* is also introduced, which is independent of *g*. The superposition of these two phase terms generates a spatially structured light field, namely, a plasmonic vortex with a topological charge *l* = *σ*(*g* − 1). During this process, the SAM *σℏ* carried by the incident CP light is converted to the plasmonic OAM by spin–orbit interaction.

**Figure 1:**
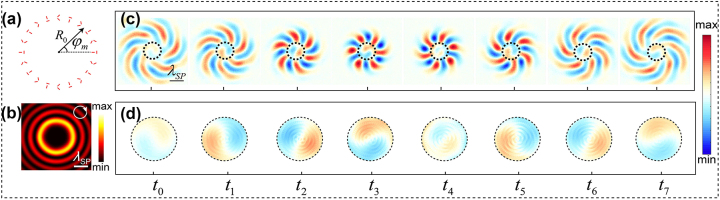
Plasmonic vortex lens and the generated plasmonic vortices. (a) Schematic of the PVL structure composed by slit-pairs arranged in a circle. (b) SP field intensity distribution in the *xy*-plane under RCP incidence. The white circle at the top-right corner denotes the spin direction of the corresponding incidence, which is similar hereinafter. (c) Snapshots of the normalized SP field amplitude evolution in the *xy*-plane, from *t*_0_ to *t*_7_ with a temporal interval of 0.5 ps. (d) Zoomed-in view of the central part (dashed circle) of (c). The more detailed evolution behaviors of this deuterogenic plasmonic vortex are provided in Supplementary Movie 1, corresponding to an interval of 0.1 ps between each frame.

We take the PVL with geometric parameters (*g*, *α*_0_) = (7, 0) shown in Figure 1a for example. Based on the 2D Huygens–Fresnel principle [42], the SP intensity distribution under RCP incidence (*σ* = 1) can be calculated and the result is displayed in Figure 1b. The field intensity exhibits a concentric ring-shaped distribution with an innermost bright ring, which can be approximately described by a Bessel function of order six, that is, $I\left(R\right)\propto {\left|J_{6}\left(k_{\text{SP}}R\right)\right|}^{2}$ , with *k*_SP_ being the SP wave number and *R* the radial coordinate. This means that a plasmonic vortex with topological charge *l*_1_ = 6 is generated, which agrees well with the theoretical expectation of *l* = *σ*(*g* − 1). However, interestingly, when we investigate the temporal evolution of the generated plasmonic vortex with the generalized 2D Huygens–Fresnel principle [34], it is noted that there is another vortex mode *l*_0_ = 1, existing at the center and concomitantly evolving with the expected outer vortex *l*_1_, as shown in Figure 1c and d. Such a vortex observed only in the time domain is quite unique and has not been reported before.

### 2.2 Decomposition of the slit-pair-based PVL structure

**Figure 2:**
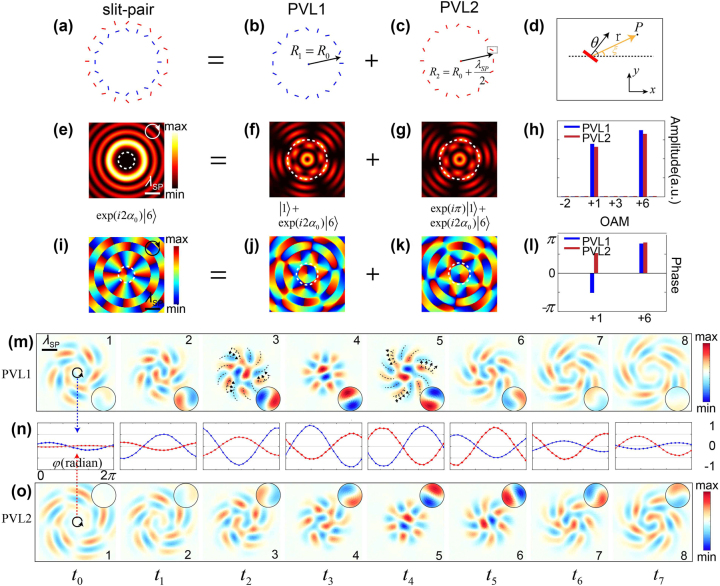
Decomposed structures of the slit-pair-based PVL and corresponding numerical results. (a–c) The slit-pair-based, inner- and outer-ring structures of the PVL shown in Figure 1a. (d) Schematic of a single slit resonator orientated by an angle of *θ* with respect to the *x*-axis. Simulations of the field intensity distributions (e–g) and phase patterns (i–k) of the generated plasmonic vortices under RCP incidence from different structures corresponding to (a–c). The white dashed circles in (f, g) label the maximum interfered intensity with a radius of 522 μm (h, l) OAM spectra of the generated plasmonic vortices. (m, o) Evolution snapshots of the normalized SP field amplitudes of PVL1 and PVL2, respectively, in the same temporal dimension as Figure 1c. Zoomed-in views of the central part are shown on the right side of the snapshots. (*n*) Generalized amplitude values extracted along the target orbit of *l*_0_ from the PVL1 (blue) and PVL2 (red) structures.

To further explain the existence of the unexpected central plasmonic vortex *l*_0_, we elaborate on the process of plasmonic vortex generation by investigating separately the performance of the inner- and outer-ring slit resonators. As demonstrated in Figure 2a–c, the inner-ring slits (blue) with *R*_1_ = *R*_0_ and the outer-ring slits (red) with *R*_2_ = *R*_0_ + *λ*_SP_/2 together form the above slit-pair PVL structure with *λ*_SP_ being the SP wavelength. Figure 2d presents a single slit resonator orientated by an angle of *θ* with respect to the *x*-axis. Here, we model the SPs excited from the slit as radiation from a dipole source directed perpendicular to its longer axis. Under normal CP incidence $E_{\text{in}}=\left(\begin{array}{cc} \sqrt{2}/2 & \sigma \text{i}\sqrt{2}/2 \end{array}\right)$ , the SP field at an arbitrary point *P* can be calculated as [43]

$$E_{P}=\eta \frac{\sqrt{2}}{2}\frac{\exp\left(\text{i}k_{\text{SP}}\left|r\right|\right)}{\text{i}\sqrt{\lambda_{\text{SP}}\left|r\right|}}\left[\frac{1}{2}{\text{e}}^{\text{i}\sigma \xi }\;+\;\frac{1}{2}{\text{e}}^{\text{i}\sigma \left(2\theta -\xi \right)}\right]$$

Here, *E*_*P*_ is the complex amplitude of the SP field at point *P*; *η* is the conversion efficiency from incident light to the SPs; **r** is the vector from the slit resonator position to point *P*; and *ξ* is the angle of **r** with respect to the *x*-axis. This demonstrates that *E*_*P*_ is comprised of two components, namely, e^i*σξ*^/2 and ${\text{e}}^{\text{i}\sigma \left(2\theta -\xi \right)}/2$ . Intriguingly, the excitation phases of these two components are both related to the azimuthal angle *ξ* with a spin dependence of *σ* and −*σ*, while the later also includes an abrupt geometrical phase shift related to *θ* with a spin dependence of 2*σ*. For the former component, it has been demonstrated that the CP light gives a phase of ±2*π* along the loop of circular slits, and hence a plasmonic vortex with the first-order Bessel function can be obtained along with spin–orbit coupling [5], corresponding to the generated OAM from the excitation component e^i*σξ*^. For the latter component, in addition to the contribution from the incident SAM, the generated OAM is also accumulated from the orientation angle variance of the slit resonators with respect to the center, which leads to the excited dipole sources varying twice faster. Combining the above two components provides the SP field excited from the circle-shaped slit structure [20]

(1) $$E\left(R,\varphi \right)\propto J_{l_{0}}\left(k_{\text{SP}}R\right){\text{e}}^{\text{i}l_{0}\varphi }+C_{1}J_{l_{1}}\left(k_{\text{SP}}R\right){\text{e}}^{\text{i}l_{1}\varphi }$$

Here, *l*_0_ = *σ* and *l*_1_ = *σ*(*g* − 1), corresponding to the generated two plasmonic vortex modes with a relative phase difference *C*_1_ = exp(2i*σα*_0_). This means the inner-ring metasurface excites the superposition of two equally-weighted OAM states with different topological charges. As an example, when PVL1 with geometric parameters (*g*, *α*_0_, *n*, *R*_1_) = (7, *π*/2, 60, 1900 μm) is illuminated by RCP incidence (*σ* = 1), the corresponding SP intensity and phase distributions are displayed in Figure 2f, j. The intensity distributions can be seen as the superposition of two OAM states with *l*_0_ = 1 and *l*_1_ = 6, which leads to the structured patterns containing one central ring (*l*_0_ = 1) surrounded by 5 ( $\left|l_{1}-l_{0}\right|$ ) outsider lobes [20], coinciding with our prediction.

For the outer-ring structure PVL2 with geometric parameters (*g*, *α*_0_, *m*, *R*_2_) = (7, *π*/2, 60, 2100 μm), due to the similar distribution of the slit resonators along the azimuthal angle *φ*_*m*_, this structure also excites two OAM modes *l*_0_ = 1 and *l*_1_ = 6, as shown in Figure 2g, k. Notably, the orientation angle difference between the *m*th slit resonators of PVL1 and PVL2 in our orthogonal slit-pair design, i.e., Δ*θ* = *θ*_1,*m*_ − *θ*_2,*m*_, is embodied in the rotation of the intensity patterns by a certain angle. Differently, considering the propagation distance of *λ*_SP_/2 due to the designed radius and the orientation angle change of –*π*/2, the excited SP field from PVL2 can be written as (see Section 2, Supplementary Materials)

(2) $$\begin{array}{ll} E\left(R,\varphi \right) & \propto J_{l_{0}}\left(k_{\text{SP}}R\right){\text{e}}^{\text{i}l_{0}\varphi }\cdot {\text{e}}^{\text{i}\pi }+C_{1}J_{l_{1}}\left(k_{\text{SP}}R\right){\text{e}}^{\text{i}l_{1}\varphi }\\ & \;\cdot {\text{e}}^{\text{i}\pi \left(1-\sigma \right)} \end{array}$$

Thus, these two structures together constitute the previously mentioned slit-pair-based PVL and the concentric ring-shaped field distribution is caused by the interference between the SPs excited from PVL1 and PVL2. Comparing Eq. (1) and Eq. (2), we can see that the interference between two rings is constructive for *l*_1_ but destructive for *l*_0_ due to the *π* phase difference at the central frequency. This leads to that only one plasmonic vortex mode with topological charge *l*_1_ = 6 can be observed in the intensity field whereas the first-order vortex *l*_0_ at the center disappears, as shown in Figures 2e and 1b. To further verify this interference, we extracted the complex SP fields *E*(*θ*) in terms of the Laguerre–Gauss basis set, respectively from PVL1 and PVL2 along the target orbit where the maximum intensity is located, as indicated by the white dashed circles in Figure 2f and g. As demonstrated in Figure 2h, *l*_0_ = +1 and *l*_1_ = +6 are the two main components in the OAM spectra of the generated plasmonic vortices for both PVL1 (blue) and PVL2 (red). Due to propagation attenuation, the amplitudes from PVL2 are slightly weaker than those from PVL1 (see Section 2, Supplementary Materials). Figure 2l illustrates the extracted phase of the above two dominant components. It can be clearly seen that, for *l*_0_ = 1, the phases from PVL1 and PVL2 are opposite with a *π* phase difference, indicating the destructive interference, which is also obvious in the central parts of their phase distributions. Whereas for *l*_1_ = 6, the complex SP fields from the two structures are in-phase. This accounts for the ignored presence of the special deuterogenic plasmonic vortex *l*_0_ in previous frequency-domain studies.

In order to further reveal the spatiotemporal dynamics of this interesting deuterogenic vortex, we investigated the temporal evolution process of the excited plasmonic vortices by tracking the formation, revolution, and decay stages of the plasmonic OAM in real time [44]. Figure 2m–o illustrate the time-resolved evolutions of the generated plasmonic vortices from the above designed PVL1 and PVL2 structures with a temporal interval of 0.5 ps, respectively. For the inner structure PVL1, in the formation stage (snapshots 1–3 in Figure 2m), the SP are excited from the slit resonators along with the spin–orbit conversion. Due to the induced geometric phase, it can be seen clearly that the initial SP excitations from all directions synchronously form 12 converging spiraling wavefront threads (the gray dashed lines) surrounding the central two threads, indicating the formation of two vortices, *l*_1_ = 6 and *l*_0_ = 1. This is followed by the concentration of the SPs into the target orbit, where the inward and outward counter-propagating SPs interfere to form the radially standing but azimuthally rotating vortex field (snapshot 4). At this revolution stage, 12 rotating lobes (6 with positive amplitude shown in orange and 6 with negative amplitude in blue) can be observed, corresponding to the steady-state e^6i*φ*^ phase of *l*_1_. The central two lobes, one with positive maximum in red and one with negative maximum in blue, correspond to the e^i*φ*^ phase distribution of *l*_0_. In the decay stage (snapshots 5–8), the SP fields form an outward-propagating spiraling wavefront and then diverge, while the handedness of the wavefront flips compared to that in the formation stage (snapshots 3 and 5), see Section 3, Supplementary Materials. It should be noted that *α*_0_ in the design determines the relative phase difference between the generated plasmonic vortices *l*_1_ = 6 and *l*_0_ = 1, and thus directly affects the temporal evolution process (see Section 4, Supplementary Materials). As indicated by Figure 2o, the temporal evolution of excited plasmonic vortices in PVL2 is roughly the same, containing the formation (snapshots 1–4), revolution (snapshot 5) and decay (snapshots 6–8) stages, except for the time delay due to the path difference Δ = *λ*_SP_/2 compared with that of PVL1.

To further study the temporally deuterogenic plasmonic vortex *l*_0_, we extracted the amplitudes of SPs on the target orbit of *l*_0_ from PVL1 and PVL2, respectively. As demonstrated in Figure 2n, 20 points were chosen uniformly along the target orbit and the generalized amplitude values were extracted. The blue curve stands for the amplitudes from PVL1 structure and the red one is from PVL2. It can be concluded that the two curves almost flip upside down, alternating with one in the peak while the other in the valley, which proves the destructive interference of *l*_0_ from the two structures on account of a *π* phase difference. However, the absolute amplitude values of the peak and valley are not exactly the same because the excitation signal is not a perfect single frequency one, and this leads to the destructively interfered vortex *l*_0_ to be observed in the temporal evolution with a weak amplitude (see Section 5, Supplementary Materials). Different from its disappearance in the intensity distribution, this temporally deuterogenic vortex *l*_0_ reflects the interaction process during the interference between PVL1 and PVL2.

In the earlier study introducing the concept of deuterogenic plasmonic vortex [35], based on the Archimedean spiral slits-based PVL, the authors rectified the ignored approximation of slit radial distance and observed that the generated vortices are compound and kaleidoscopic. In contrast, our work utilized a slit-pair-based PVL, where both the inner- and outer-rings excite two plasmonic vortex mode, *l*_0_ and *l*_1_. Through properly designing the separation distance, one mode undergoes instructive interference but the other interferes destructively. Nevertheless, the destructively interfered mode can also be observed in temporal evolution. Consequently, our proposed deuterogenic vortex mode is a temporal one, which can be observed only in the time domain and interferes destructively in the intensity field.

### 2.3 Experimental verification

**Figure 3:**
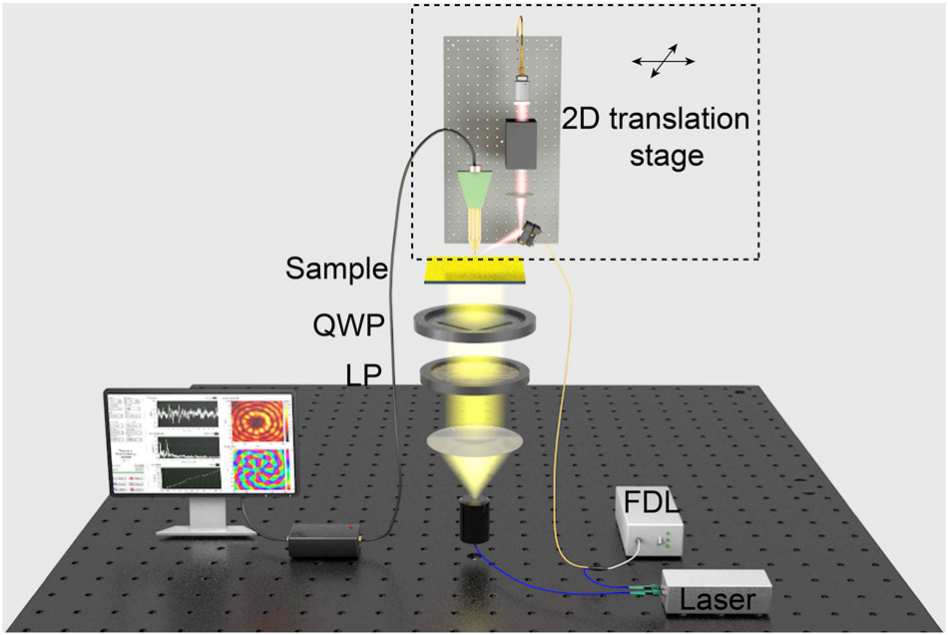
Schematic of the experimental NSTM setup to detect the vertical component of the SP field Ez. LP, Linear polarizer; QWP, Quarter-wave plate; FDL, Fiber-based delay line.

As proof-of-concept experiments, we fabricated samples working at 0.75 THz and characterized the generated plasmonic vortices using a fiber-based NSTM system. As schematically shown in Figure 3, the in-fiber femtosecond source is split into two beams, one used to generate THz radiation (blue) and the other to detect SP fields (yellow). In the transmitter modules, the femtosecond beam with a fiber-based delay line (FDL) first illuminates onto a commercial photoconductive antenna to generate broadband THz radiation. Other devices, such as a THz lens, a polarizer and a quarter-wave plate are placed between the antenna and the sample to generate the target incident beam and excite SPs. The other beam illuminates onto a commercial THz near-field probe [45], in which the movement of excited carriers forms a current proportional to the detected THz SP field (see methods). The basic idea of NSTM is to temporally record the waveform, step by step, by changing the FDL to sample the SP field at different time-delays and eventually obtain the whole time-domain signal. This direct measuring and imaging process gives access to full amplitude and phase information with subwavelength spatial resolution and deep sub-optical-cycle temporal resolution.

**Figure 4:**
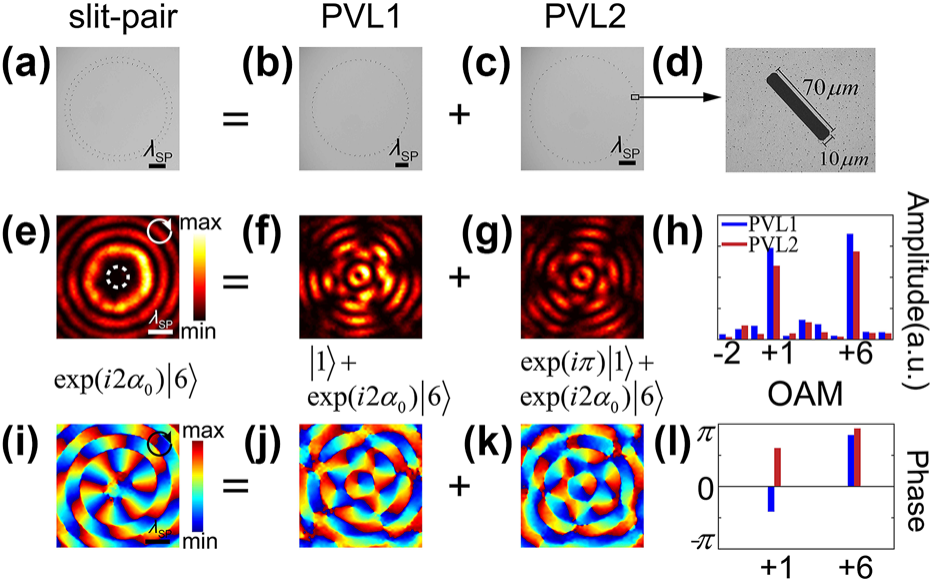
Microscopic images of the fabricated samples and experimental results. (a–c) Microscopic images of the fabricated samples composed by identical slit resonators. (a) PVL structure based on 60 slit-pairs. (b) PVL1 structure with *R*_1_ = 1900 μm. (c) PVL2 structure with *R*_2_ = 2100 μm. (d) Fabricated slit with geometric parameters: Width = 10 μm and length = 70 μm. Experimental results of the field intensity distributions (e–g) and phase patterns (i–k) of the generated plasmonic vortices corresponding to PVLs (a–c) under RCP incidence, respectively. (h, l) Extracted OAM spectra.

Figure 4 illustrates the microscopic images of the fabricated samples (Figure 4a–d) and the experimental near-field results of the SP field intensity (Figure 4e–g) and phase (Figure 4i–k) distributions in the frequency domain under RCP incidence at 0.75 THz. Clearly, these results agree well with the numerical investigations shown in Figure 2. Both PVL1 and PVL2 excited two equally weighted plasmonic vortex modes *l*_0_ = 1 and *l*_1_ = 6. In the slit-pair-based PVL, the small bright intensity ring corresponding to *l*_0_ vanished (indicated by the white dashed circle in Figure 4e), leaving only the outer brightest ring, whose radius corresponded to the first extremum of the Bessel function of *l*_1_. To see the relative intensity of the individual OAM modes more clearly, we decomposed the complex field in terms of the Laguerre–Gauss basis set along the target orbit where the maximum intensity is located. As demonstrated in Figure 4h, the OAM modes *l*_0_ = 1 and *l*_1_ = 6 were the two dominant components. The phase of the extracted field in Figure 4l indicates destructive interference for the former but constructive for the latter, verifying our explanation.

**Figure 5:**
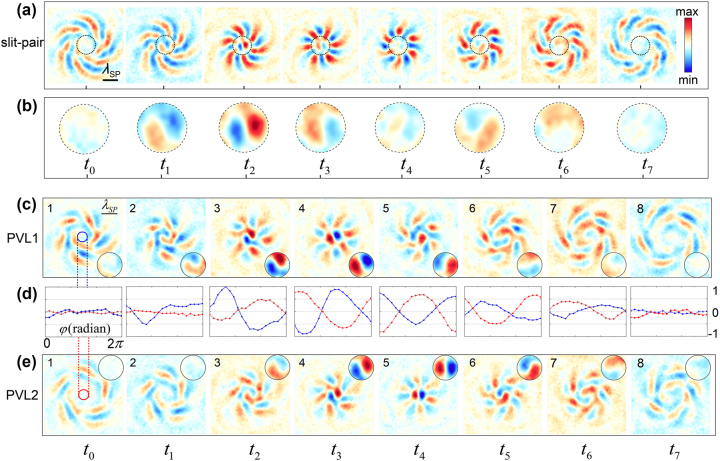
Measured time-resolved snapshots of the SP field based on the NSTM system, generated from the slit-pair-based PVL (a), inner PVL1 (c) and outer PVL2 (e) at an interval of 0.5 ps. (b) Zoomed-in views of the central part of (a). (d) Generalized amplitude values extracted from PVL1 (blue) and PVL2 (red)

Figure 5 illustrates the experimentally measured spatiotemporal evolution of the generated plasmonic vortices in the above three PVLs with at an interval of 0.5 ps, identical with that in the simulation. Here, we show the time-resolved snapshots of the SP fields in the *xy*-plane and the extracted absolute SP amplitudes on the target orbit of *l*_0_. To recognize the temporally deuterogenic vortex *l*_0_, zoomed-in views of the central part in the SP field are also provided in Figure 5b. Although the interference for *l*_0_ is destructive, one can still observe the two rotating lobes at the center besides the outer 12 converging and spiraling wavefront threads, as numerically predicted in Figure 1. According to Figure 5c–e, both PVLs excite two equally weighted plasmonic vortices with topological charges *l*_0_ = 1 and *l*_1_ = 6, covering the formation, revolution, and decay stages in the lifetime, which verified the whole interference process. In addition to the fabrication- and measurement-induced errors, the deviations between numerical and experimental results can be mainly attributed to the slight difference between the numerical and actual SP waveforms. Nevertheless, the experimental results complied well with those from the numerical investigation, and experimentally verified the existence of the temporally deuterogenic plasmonic vortex with high temporal and spatial resolutions, which is accessible only in the time-domain investigation.

Compared with traditional time-resolved measurement techniques such as the two-photon photoemission electron microscopy [44] which mainly rely on the interference between light in the signal and reference branches to achieve characterization, the NSTM adopted in our work can directly detect the electric components perpendicular to the surface, making it possible to acquire more exact evolution dynamics of surface plasmons. In addition, the full amplitude and phase information can be simultaneously and accurately measured which allows us to investigate the deeper mechanism of the constructive or destructive interferences between plasmonic vortices. It should be emphasized that since the waveforms were experimentally obtained with consecutive time steps of 20 fs in our system, it is possible to reveal the plasmonic vortex evolution in deeper sub-optical-cycle temporal resolution. Supplementary Movie 2 shows a more complete and detailed evolution process with the interval between each frame corresponds to 20 fs in experimental results.

## 3 Discussion

So far, we have numerically explained and experimentally verified the fact that the deuterogenic plasmonic vortex mode *l*_0_ appeared only in the time domain along with plasmonic spin–orbit interaction, which reveals an exotic spin–orbit coupling phenomenon. Previous studies have not reported this phenomenon due to the destructive interference of *l*_0_ at the central frequency, whenever the slit-pair is separated by an odd multiple of *λ*_SP_/2. It is worth mentioning that, when the two slits are separated by quite a large distance, the deuterogenic vortex mode would be recognizable in the intensity pattern due to the obvious amplitude difference between PVL1 and PVL2. Nevertheless, these structures lose the feasibility and generality for both basic researches and practical applications (see Section 6, Supporting Information). Actually, the pulsed pump light, rather than a continuous single-frequency light source, allows us to observe the existence of the deuterogenic plasmonic vortex in temporal evolution process. In fact, with the rapid development of femtosecond lasers, many fundamental light science research and future applications require pulsed light excitation. Therefore, our research on the unique temporally deuterogenic vortex is of great importance for many practical applications related to time-varying characteristics. One of the most appropriate occasions is plasmonic rotators. Due to the existence of the central plasmonic vortex, real-time rotating of multiple particles can be realized through reasonable designs since the temporally existing vortex modes, *l*_0_ and *l*_1_, are endowed with different angular velocities and orbits, providing different scattering forces [46]. This allows the rotation speed and radius of the controlled particles to be designed on demand by manipulating the topological charges of the excited plasmonic vortices. In addition, the rotation direction can be flexibly flipped depending on the chirality of the incident beams (see Section 7, Supporting Information). Compared with traditional optical rotators, our design based on the plasmonic vortices can achieve manipulation with higher degrees of freedoms and on a scale smaller than the diffraction limit.

## 4 Conclusions

In summary, we reported the existence of a unique temporally deuterogenic plasmonic vortex mode during spin–orbit coupling and revealed its spatiotemporal distribution and the corresponding evolution of the plasmonic OAM. This deuterogenic vortex can be tailored with different PVL designs and vortex beam incidences. The general principle not only helps us understand the intrinsic nature of plasmonic spin–orbit interactions, but also presents a novel avenue to manipulate compound vortex modes. The unusual evolution process forms a special optical force distribution in both spatial and temporal domains, which stimulates new research on the mechanism and potential applications of on-chip integrated devices. In addition, the proposed plasmonic OAM manipulation strategy is general and can be directly applied to the infrared and visible regimes. Thus, this exotic spin–orbit coupling phenomenon is expected to render new effects when they are exploited in light–matter interactions.

## 5 Methods

### 5.1 Sample fabrication

The samples were fabricated by conventional photolithography, metallization, and spin-coating processing. First, thermal evaporation was employed to deposit a 200 nm**-**thick aluminum film on a 2 mm-thick double-side polished high-resistivity silicon wafer. Then a layer of photoresist (AZ P4000) was spin-coated on the aluminum film. The patterns for the plasmonic vortex couplers were exposed by standard lithography. The photoresist and aluminum in the exposed area were removed by development and etching processes, respectively. Finally, the remaining photoresist in other area was washed away by acetone, leaving the aluminum film perforated with the desired structures on the silicon wafer. To enhance the confinement of the SPs on the metal surface, a 10 μm-thick polyimide layer was coated on the whole structure. The dimension of the slit resonators was set to be 70 μm × 10 μm for a working frequency of 0.75 THz, corresponding to *λ*_SP_ ≈ 400 μm.

### 5.2 Measurement

A fiber-based NSTM system was employed to experimentally generate the incident THz radiation and detect the generated plasmonic vortices. Femtosecond fiber laser output with ∼50 fs pulse width and 1550 nm central wavelength is split into two beams. After passing through a fiber-based delay line, one of the beams is used in the transmitter modules to illuminate a commercial photoconductive antenna and excite electron–hole pairs on its surface. Under an external voltage, these carriers will accelerate and generate a transient photocurrent, producing broadband THz radiation. The generated THz radiation is collimated by a THz lens and passes through a linear polarizer (LP) to form a linearly polarized THz beam. Through adjusting the angle of the subsequent 1/4 wave plate with respect to the LP, experimental results under CP incidence can be obtained. In the detector modules, the other beam is converted to free space light by a fiber collimator and then sent through a frequency doubling crystal (BBO). Focused by a lens and then reflected by a mirror, it ultimately illuminates onto the commercial THz near-field probe. The THz probe is fixed on a 2D electrically controlled translation stage to scan and obtain the full amplitude and phase information of SP fields with a high spatial resolution. In our experiment, all the measurements were obtained at a plane about 75 μm above the sample surface, and the scanned range was set to be 2 mm × 2 mm with a 40 μm step. The sampling step was chosen as 20 fs corresponding to ∼1/66 of the optical-cycle at 0.75 THz.

## Abbreviations

OAM: orbital angular momentum
SPs: surface plasmons
SAM: spin angular momentum
NSTM: near-field scanning terahertz microscopy
PVLs: plasmonic vortex lenses
CP: circularly polarized
FDL: fiber-based delay line

## Supplementary Material

Supplementary Material Details

## References

[1] Shen Y. (2019). Optical vortices 30 years on: OAM manipulation from topological charge to multiple singularities. *Light Sci. Appl.*.

[2] Prinz E., Hartelt M., Spektor G., Orenstein M., Aeschlimann M. (2023). Orbital angular momentum in nanoplasmonic vortices. *ACS Photon.*.

[3] Wang X., Nie Z., Liang Y., Wang J., Li T., Jia B. (2018). Recent advances on optical vortex generation. *Nanophotonics*.

[4] Zang X. (2021). Metasurfaces for manipulating terahertz waves. *Light: Adv. Manuf.*.

[5] Kim H., Park J., Cho S. W., Lee S. Y., Kang M., Lee B. (2010). Synthesis and dynamic switching of surface plasmon vortices with plasmonic vortex lens. *Nano Lett*..

[6] Bai Y., Yan J., Lv H., Yang Y. (2022). Plasmonic vortices: a review. *J. Opt.*.

[7] Gorodetski Y., Niv A., Kleiner V., Hasman E. (2008). Observation of the spin-based plasmonic effect in nanoscale structures. *Phys. Rev. Lett*..

[8] Shitrit N., Bretner I., Gorodetski Y., Kleiner V., Hasman E. (2011). Optical spin Hall effects in plasmonic chains. *Nano Lett*..

[9] Zhang X. (2020). Terahertz surface plasmonic waves: a review. *Adv. Photon.*.

[10] Xu Q. (2023). Meta-optics inspired surface plasmon devices. *Photon. Insights*.

[11] Pan W. (2022). High-efficiency generation of far-field spin-polarized wavefronts via designer surface wave metasurfaces. *Nanophotonics*.

[12] Chen Y. (2023). Efficient meta-couplers squeezing propagating light into on-chip subwavelength devices in a controllable way. *Nano Lett.*.

[13] Spektor G. (2019). Mixing the light spin with plasmon orbit by nonlinear light-matter interaction in gold. *Phys. Rev. X*.

[14] Jacob Z., Shalaev V. M. (2011). Plasmonics goes quantum. *Science*.

[15] Erhard M., Fickler R., Krenn M., Zeilinger A. (2018). Twisted photons: new quantum perspectives in high dimensions. *Light Sci. Appl.*.

[16] Guan F. (2023). Overcoming losses in superlenses with synthetic waves of complex frequency. *Science*.

[17] Min C. (2013). Focused plasmonic trapping of metallic particles. *Nat. Commun.*.

[18] Tsai W. Y., Huang J. S., Huang C. B. (2014). Selective trapping or rotation of isotropic dielectric microparticles by optical near field in a plasmonic archimedes spiral. *Nano Lett*..

[19] Zaman M. A., Padhy P., Hesselink L. (2019). Solenoidal optical forces from a plasmonic Archimedean spiral. *Phys. Rev. A*.

[20] Zhang Y. (2019). Manipulation for superposition of orbital angular momentum states in surface plasmon polaritons. *Adv. Opt. Mater.*.

[21] Tsai W. Y. (2019). Twisted surface plasmons with spin‐controlled gold surfaces. *Adv. Opt. Mater.*.

[22] Zang X. (2019). Manipulating terahertz plasmonic vortex based on geometric and dynamic phase. *Adv. Opt. Mater.*.

[23] Prinz E., Spektor G., Hartelt M., Mahro A. K., Aeschlimann M., Orenstein M. (2021). Functional meta lenses for compound plasmonic vortex field generation and control. *Nano Lett*..

[24] Tan Q., Guo Q., Liu H., Huang X., Zhang S. (2017). Controlling the plasmonic orbital angular momentum by combining the geometric and dynamic phases. *Nanoscale*.

[25] Zang X. (2020). Geometric metasurface for multiplexing terahertz plasmonic vortices. *Appl. Phys. Lett.*.

[26] Ahmed H. (2022). Optical metasurfaces for generating and manipulating optical vortex beams. *Nanophotonics*.

[27] Zhu Y., Zang X., Chi H., Zhou Y., Zhu Y., Zhuang S. (2023). Metasurfaces designed by a bidirectional deep neural network and iterative algorithm for generating quantitative field distributions. *Light: Adv. Manuf.*.

[28] Cho S.-W., Park J., Lee S.-Y., Kim H., Lee B. (2012). Coupling of spin and angular momentum of light in plasmonic vortex. *Opt. Express*.

[29] Cui T., Sun L., Bai B., Sun H. B. (2021). Probing and imaging photonic spin–orbit interactions in nanostructures. *Laser Photon. Rev.*.

[30] Devlin R. C., Ambrosio A., Rubin N. A., Mueller J. B., Capasso F. (2017). Arbitrary spin-to-orbital angular momentum conversion of light. *Science*.

[31] Chong A., Wan C., Chen J., Zhan Q. (2020). Generation of spatiotemporal optical vortices with controllable transverse orbital angular momentum. *Nat. Photon.*.

[32] Chen W., Liu Y., Lu Y.-Q. (2023). Spatiotemporal optical vortices: toward tailoring orbital angular momentum of light in full space-time. *ACS Photon.*.

[33] Spektor G., Prinz E., Hartelt M., Mahro A.-K., Aeschlimann M., Orenstein M. (2021). Orbital angular momentum multiplication in plasmonic vortex cavities. *Sci. Adv.*.

[34] Yuan X. (2023). Tailoring spatiotemporal dynamics of plasmonic vortices. *Opto-Electron. Adv.*.

[35] Yang Y. (2020). Deuterogenic plasmonic vortices. *Nano Lett*..

[36] Li S. (2020). Helicity-delinked manipulations on surface waves and propagating waves by metasurfaces. *Nanophotonics*.

[37] Lang Y. (2022). On-chip plasmonic vortex interferometers. *Laser Photon. Rev.*.

[38] Zhang X. (2015). Anomalous surface wave launching by handedness phase control. *Adv. Mater*..

[39] Lin J. (2013). Polarization-controlled tunable directional coupling of surface plasmon polaritons. *Science*.

[40] Xu Q. (2016). Polarization-controlled surface plasmon holography. *Laser Photon. Rev.*.

[41] Pan W. (2023). Efficiently controlling near-field wavefronts via designer metasurfaces. *ACS Photon.*.

[42] Teperik T. V., Archambault A., Marquier F., Greffet J. J. (2009). Huygens–Fresnel principle for surface plasmons. *Opt. Express*.

[43] Jiang X. (2023). Geometric phase control of surface plasmons by dipole sources,” *Laser Photon*. *Rev.*.

[44] Spektor G. (2017). Revealing the subfemtosecond dynamics of orbital angular momentum in nanoplasmonic vortices. *Science*.

[45] Wächter M., Nagel M., Kurz H. (2009). Tapered photoconductive terahertz field probe tip with subwavelength spatial resolution. *Appl. Phys. Lett.*.

[46] Zhang Y. (2021). Plasmonic tweezers: for nanoscale optical trapping and beyond. *Light Sci. Appl.*.

