# Supplementary material for: Temporally deuterogenic plasmonic vortices
Source: Nanophotonics. 2024 Feb 21;13(6):955–63. doi: 10.1515/nanoph-2023-0931 (PMC11614328; doi:10.1515/nanoph-2023-0931)
Supplement: Supplementary file 1 — Supplementary Material Details [file j_nanoph-2023-0931_suppl_001.docx]

Supplementary Materials

**Temporally Deuterogenic Plasmonic Vortices**

This file includes:

Section 1: The plasmonic vortex lens design.

Section 2: Surface plasmon field excited in the PVL2 structure.

Section 3: The handedness flip of the SPs spiraling wavefront between the formation and decay stages.

Section 4: Spatiotemporal evolutions with different *α*_0_ designs in PVL1.

Section 5: The influence of slit-resonator resonance response on the generated temporally deuterogenic plasmonic vortex.

Section 6: Numerical investigations of the PVL with slit-pair separated by *λ*_SP_, 3*λ*_SP_/2 and larger distances.

Section 7: Generated plasmonic vortices in slit-pair-based PVL under different incidences.

Additional supplementary movies:

Supplementary Movie 1:

Detailed evolution behaviors of the plasmonic vortices generated by slit-pair-based PVL, corresponding to the results of Figure 1c, at an interval of 0.1 ps between each frame.

Supplementary Movie 2:

The measured time-resolved evolution process of the plasmonic vortices generated by slit-pair-based PVL under RCP incidence, corresponding to the results of Figure 5a, with the interval between each frame corresponds to 20 fs in experimental results.

**Section 1: The plasmonic vortex lens design.**

The plasmonic vortex lens consists of uniformly rotated slit-resonator-pairs arranged in a circular ring with radius *R*_0_ as shown in Figure S1. All slit resonators have the same geometric dimension but different orientation angles described as *θ*_1_, *_m_* and *θ*_2_, *_m_* for the inner- and outer-ring slits, respectively.

**
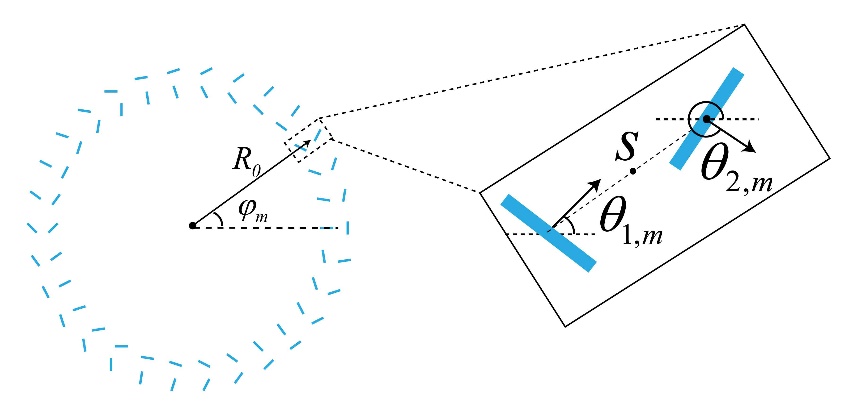
**

**Figure S1.** Schematics of the plasmonic vortex lens design. Here, *θ*_1_, *_m_* and *θ*_2_, *_m_* represents the orientation angle of the inner- and outer-slit resonator of the *m*th slit-pair, respectively. *φ_m_* is the azimuthal angle of the *m*th slit-pair with respect to the *x*-axis. *R*_0_ corresponds to the radius the of the plasmonic vortex lens.

**Section 2: Surface plasmon field excited in the PVL2 structure.**

For the slit resonators in PVL2, considering the prorogation distance *λ*_SP_/2 between the inner- and outer-ring, the SP field at the field point *P* can be calculated as

$$E_{P}=\eta\frac{\sqrt{2}}{2}\frac{\exp\left(\text{i}k_{\text{SP}}\left|\mathbf{r}'\right|\right)}{\text{i}\sqrt{\lambda_{\text{SP}}\left|\mathbf{r}'\right|}}\left[\frac{1}{2}e^{\text{i}\sigma\xi}+\frac{1}{2}e^{\text{i}\sigma(2\theta_{2,\text{m}}-\xi)}\right]$$

$E_{P}=\eta\frac{\sqrt{2}}{2}\frac{\exp\left(\text{i}k_{\text{SP}}\left|\mathbf{r}\right|\right)}{\text{i}\sqrt{\lambda_{\text{SP}}\left|\mathbf{r}'\right|}}\left[\frac{1}{2}e^{\text{i}\sigma\xi}+\frac{1}{2}e^{\text{i}\sigma(2\theta_{2,\text{m}}-\xi)}\right]\cdot e^{\text{i}k_{\text{SP}}\lambda_{\text{SP}}/2}$. (S1)

Considering the relation of *θ*_2_, *_m_* = *θ*_1_, *_m_* – π/2 provides

$E_{P}=\eta\frac{\sqrt{2}}{4}\frac{\exp\left(\text{i}k_{\text{SP}}\left|\mathbf{r}\right|\right)}{\text{i}\sqrt{\lambda_{\text{SP}}\left|\mathbf{r}'\right|}}\left(e^{\text{i}\sigma\xi}+e^{\text{i}\sigma(2\theta_{1,\text{m}}-\pi-\xi)}\right)\cdot e^{\text{i}k_{\text{SP}}\lambda_{\text{SP}}/2}$.

From Eq. S1, it can be also concluded that due to the propagation attenuation between$\mathbf{r}$and $\mathbf{r}'$, the amplitudes in the PVL2 are slightly weaker than that from the PVL1.

Section 3: The handedness flip of the SPs spiraling wavefront between the formation and decay stages.

The entire evolution process of generated plasmonic vortices comprises three stages: formation, revolution, and decay. During the formation stage, surface plasmons (SPs) are excited from the slit resonators along with the spin−orbit conversion. Owing to the induced geometric phase, the excited SPs exhibit a phase gradient and form converging spiraling wavefront threads. For clarity and intuitive observation, we consider the example of a single-ring PVL with geometric parameters (*g*, *α*_0_, *R*_2_) = (5, π/2, 2000 μm), which excites plasmonic vortex with topological charge *l*_0_ = 4 under RCP incidence. Here, we study the phase distribution on a quarter of the PVL. As illustrated in Figure S2a, the outer orange dashed circle and inner purple dashed circle represent the PVL structure and the target orbit, respectively. The five solid black circles depict the phase of the excited SPs. Due to the induced phase gradient, the five sources generate waveforms with an initial phase difference of 2π. Thus, the five colorful points determine the handedness of the SPs spiraling wavefront. With the inward propagation of the wavefront, the five points get closer and gradually converge on the target orbit (Figure S2b-e). In the decay stage, five points deviate from the revolution orbit and propagate outward. At this moment, the handedness of the SPs spiraling wavefront flips (Figure S2f-g). It is worth mentioning that the whole evolution process of the spiraling wavefronts is general and can be applied to other plasmonic vortices with different topological charge.


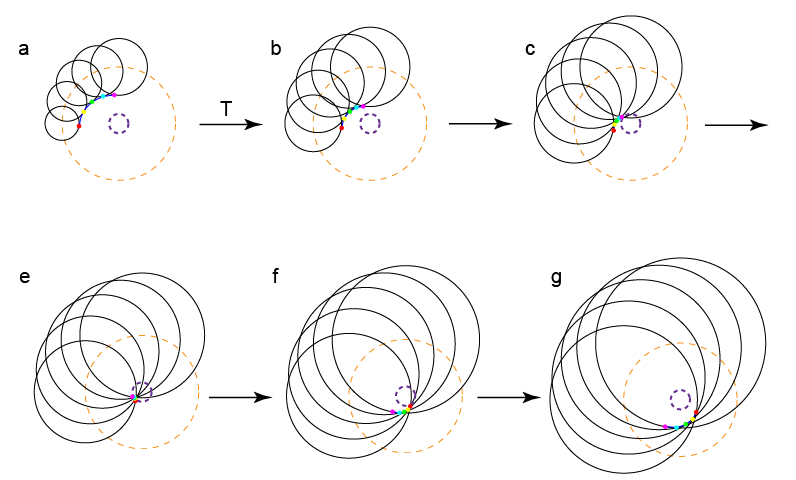


Figure S2. Snapshots of the SP field wavefront evolution under RCP incidence with a temporal interval of one optical-cycle at 0.75 THz.

**Section 4: Spatiotemporal evolutions with different *α*_0_ designs in PVL.**

Since the *α*_0_ in PVL design determines the relative phase difference between excited plasmonic vortices *l*_0_ and *l*_1_ as demonstrated by Eq. 1 in the manuscript, it directly affects the spatiotemporal dynamics process of the generated vortices. Here, we investigate the detailed temporal evolutions of the excited plasmonic vortices under different *α*_0_ designs, with *α*_0_ = 0, π/4, π/2 and 3π/4, respectively. As demonstrated by Figure S3, varying *α*_0_ leads to different initial excitation phase of *l*_1_. In addition, the distribution of central two lobes at the same moment keeps invariable since the plasmonic vortex *l*_0_ is independent on the orientation angles of the slit resonators, corresponding to the component of $e^{\text{i}\sigma\xi}/2$. It should be noted that the results of *α*_0_ = π is the same with that of *α*_0_ = 0 due to the $e^{2i\sigma\alpha_{0}}$phase difference. To contrast this variation more clearly, we choose 60 points uniformly along the target orbit of *l*_1_ labeled by the black dashed circle in Figure S3a and extract their generalized amplitude values from *t*_0_ to *t*_2_ at an interval of 0.5 ps, as shown in Figure S3b. It can be concluded that different *α*_0_ designs correspond to different field distributions of *l*_1_ at the same moment.

**
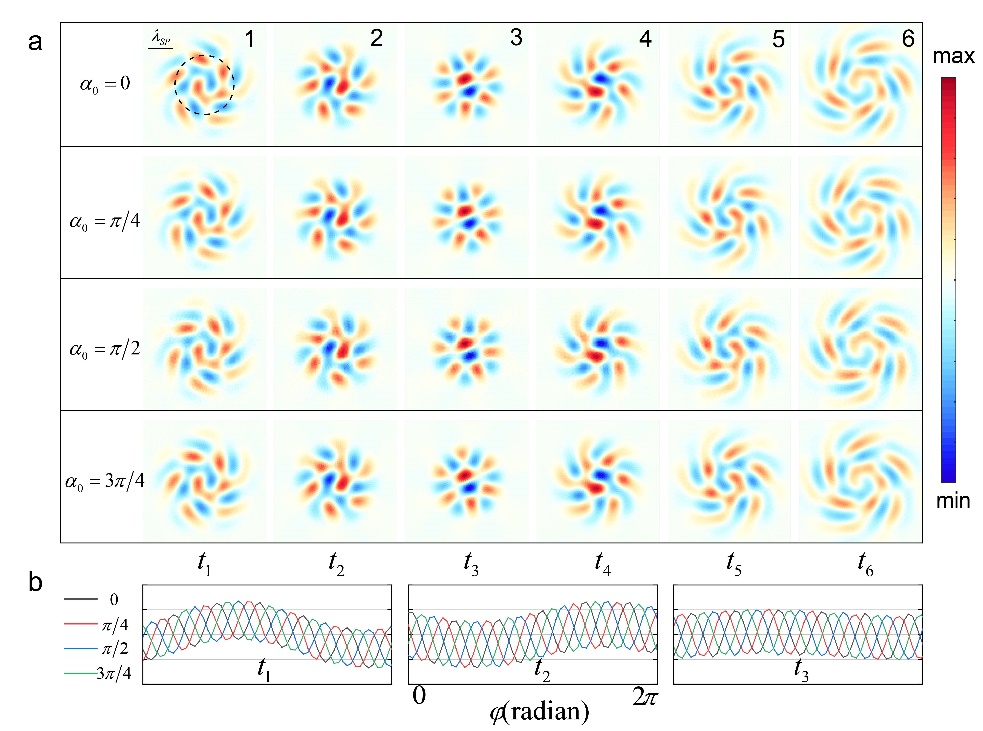
**

**Figure S3.** (a) Time-resolved evolutions of the excited plasmonic vortices under different *α*_0_ designs from *t*_0_ to *t*_5_ at an interval of 0.5 ps, identical with that in the manuscript. (b) Generalized amplitude values extracted along the target orbit.

**Section 5: The** **influence of slit-resonator resonance response** **on** **the generated temporally deuterogenic plasmonic vortex.**

Since the resonance response of slit resonator directly determines the waveform of the excited SPs, it can also tailor the spatiotemporal dynamics of the generated temporally deuterogenic plasmonic vortex. Here, we take two different waveforms, shown in Figures S4a and S4b for example, corresponding to the one employed in the manuscript and a higher Q resonance than that, respectively. The PVL design is identical with that in the manuscript with (*g*, *α*_0_, *n*, *R*_1_, *R*_2_) = (7, π/2, 60, 1900 μm, 2100μm), exciting two plasmonic vortex modes *l*_1_ = 6 and *l*_0_ = 1. We carried out numerical investigation on the SPs filed evolution and the results are shown in Figures S4d and S4e, respectively.

It can be seen that for a higher Q resonance (Waveform 2), the revolution stage continues longer (snapshots 5–7) due to the more oscillations of the waveform. In addition, the difference of absolute amplitude values between the peak and valley becomes slighter and this makes the central deuterogenic plasmonic vortex less recognizable. However, on can still observe the temporally concomitant plasmonic vortex in the formation and decay stages. This means, when we illuminate the slit-pair-based PVL using a single-frequency signal, the interference of deuterogenic vortex *l*_0_ will be completely destructive, even in the temporal evolution due to the exactly same value but opposite sign of the amplitude from the inner PVL1 and outer PVL2 structures.


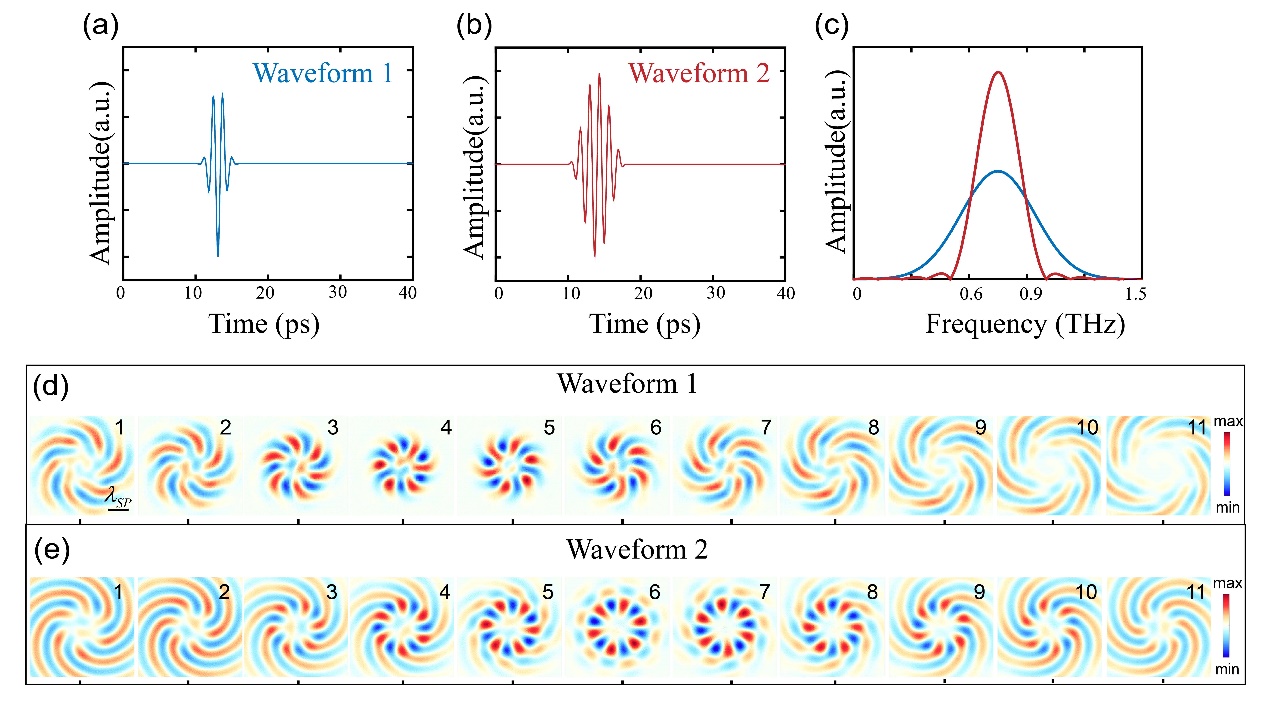


**Figure S4.** (a) The excitation waveform 1 of slit resonator resonance employed in the manuscript. (b) Waveform 2 possessing higher Q than that in the manuscript. (c) Fourier transformation results of the time-domain signals, waveform 1 in blue and waveform 2 in red, with Q1 = 0.4546 and Q2 =1.5698. Time-resolved evolutions of the generated plasmonic vortices with waveform 1 (d) and higher Q waveform 2 (e) under RCP incidence in the slit-pair-based PVL, respectively.

In addition, we also investigate the temporal evolution of the generated plasmonic vortices under single frequency signal *f* = 0.75 THz incidence. As shown in Figure S5, the central *l*_0_ = 1 can only be observed in the formation stage (snapshots 1-3), which corresponds to the beginning of the resonance signal. Afterwords, the inward and outward counter-propagating SPs interfere to form the radially standing but azimuthally rotating vortex field (snapshots 4-6) and then keeps in the steady state (snapshots 7-10). In the steady state, only rotating twelve lobes can be observed and throughout the whole evolution process, the deuterogenic vortex *l*_0_ totally disappears due to the completely destructive interference.

**
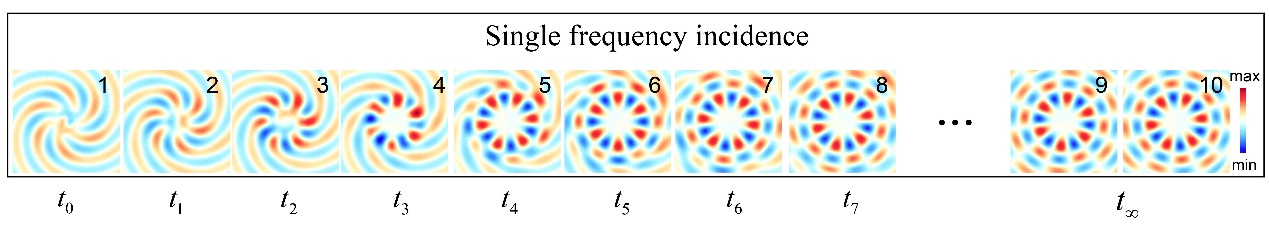
**

**Figure S5.** Time-resolved evolutions of the generated plasmonic vortices under single frequency waveform incidence in the slit-pair-based PVL at an interval of 0.5 ps.

**Section 6:** **Numerical investigation of the plasmonic vortex lens with slit-pair separated by *λ*_SP_, 3*λ*_SP_/2 and larger distances.**

We have numerically explained and experimentally verified the deuterogenic plasmonic vortex *l*_0_ appeared only in the time domain, which reveals an exotic spin-orbit coupling phenomenon. Previous studies failed to report this phenomenon due to destructive interference of *l*_0_ at the central frequency, whenever the slit-pair is separted by odd multiple of *λ*_SP_/2. However, if the two slit resonators of the PVL are separated by a distance of *λ*_SP_, that is, the path difference is even multiple of *λ*_SP_/2, the temporal evolution process will be totally different. In this condition, the SPs field excited from the PVL1 structure is similar with that in the manuscript, described as

. (S2)

Since there is a path difference of *λ*_SP_, the SPs field excited from the outer-ring structure becomes

. (S3)

Comparing Eq. S2 and S3, the SPs field from two structures form instructive interference for *l*_0_ since the phase difference is 2π. Whereas for *l*_1_ = 6, the interference is destructive, leaving only the small light ring in the intensity pattern, as shown in Figure S6a. We also extracted the complex SP fields *E*(*θ*) in terms of the Laguerre-Gauss basis set, from the inner- and outer-ring along the target orbit where the maximum interference intensity is located, corresponding to the radius of 522 μm, same with that in the manuscript. As demonstrated in Figure S6c and 6d, in the OAM spectra, the phases of *l*_1_ = 6 component from PVL1 and PVL2 have a π difference, verifying the destructive interference. In this condition, *l*_1_ = 6 becomes the deuterogenic plasmonic vortex mode and it can be observed only in the temporal evolution snapshots, as shown in Figure S6e.


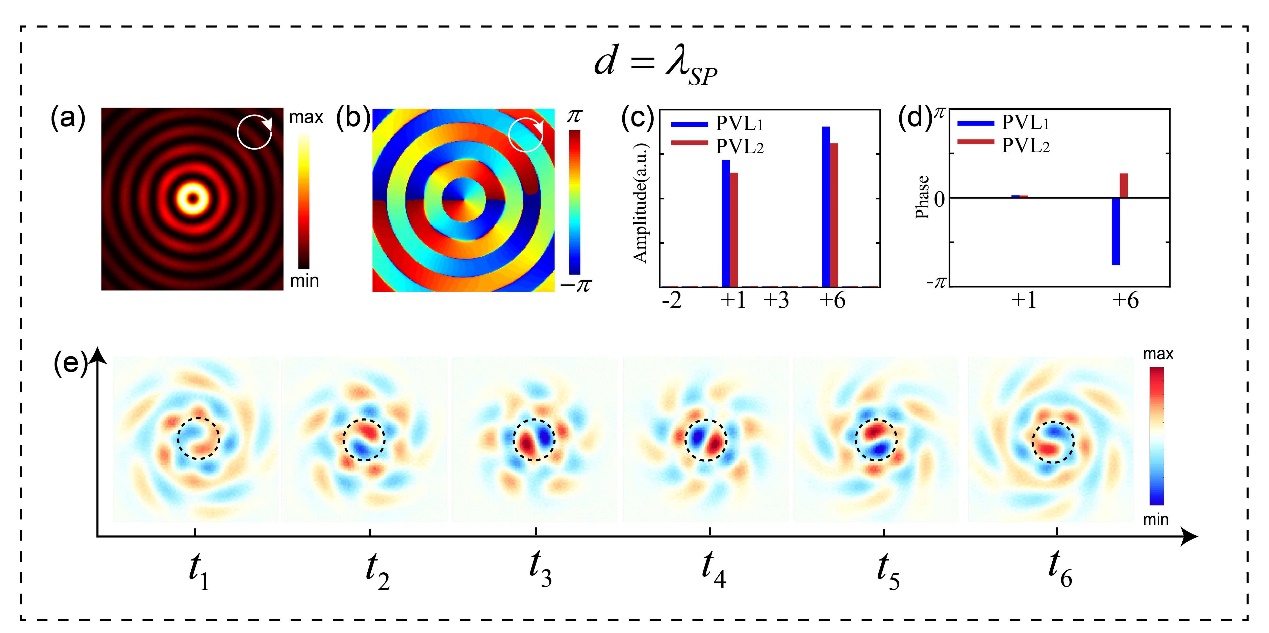


**Figure S6.** Numerical results of the PVL with slit-pair separated by *λ*_SP_. (a, b) The intensity and phase distributions of SP fields under RCP incidence. (c, d) OAM spectra of the generated PVs. (e) Snapshots of the normalized SP amplitude field evolution in the *xy*-plane, from *t*_0_ to *t*_5_ at a temporal interval of 0.5 ps.

In order to explore more design degrees of freedom, we separated the two slit resonators in the PVL by a distance of 3*λ*_SP_/2, that is, the path difference is odd multiple of *λ*_SP_/2. In this condition, the SPs field excited from the inner-ring PVL1 structure is the same with that in the manuscript, while the SPs field excited from the outer-ring PVL2 structure becomes

.

It can be concluded that the SPs field from two structures forms the instructive interference for *l*_1_ but destructive for *l*_0_, similar with the separation of *λ*_SP_/2. However, due to the larger propogation distance, the excited SPs field from inner and outer structure are separated more distinctly in the temporal evolution, corresponding to a longer evolution process, as shown in Figure S7. Compared with the separation of *λ*_SP_/2, where the strong temporal overlaps lead to the weak ampitude of *l*_0_, in this case, both *l*_0_ and *l*_1_ show analogous field intensity and the temporally deuterogenic vortex mode *l*_0_ can be observed obviously. In this way, the seperation distance can serve as another design degree of freedom to manipulate the spatiotemporal dynamics of the deuterogenic plasmonic vortices, not only for the switch of instructive and destructive interference, but also the overlap in the temporal evolution.


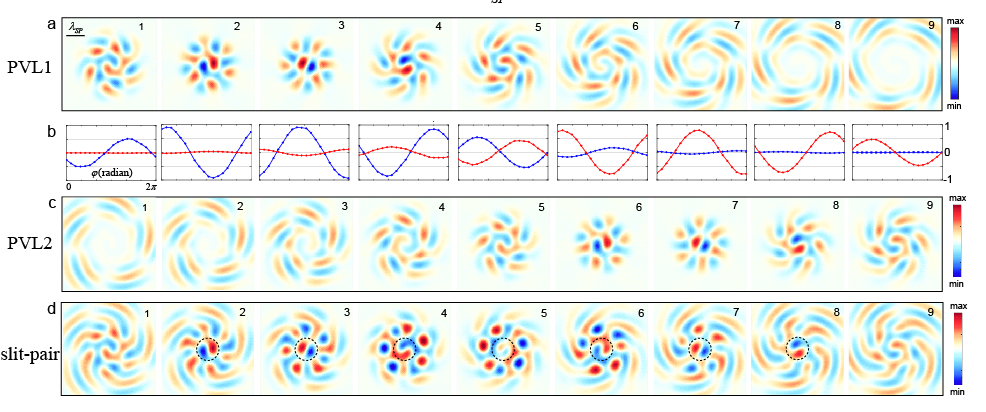


**Figure S7.** Time-resolved evolutions of the generated plasmonic vortices with the slit-pair separated by 3*λ*_SP_/2. (a, c, d) Snapshots of the normalized SP amplitude field evolution in the *xy*-plane under RCP incidence in PVL1, PVL2 and slit-pair-based PVL, respectively. (b) Generalized amplitude values extracted from the PVL1 (blue) and PVL2 (red) structures.

Analysis of Figure 2h in the manuscript reveals that, owing to propagation attenuation, the amplitudes from PVL2 are slightly weaker than those from PVL1 for both modes *l*_0_ = 1 and *l*_1_ = 6. Therefore, it is reasonable that the deuterogenic vortex mode would be observed resulting from the obvious amplitude difference between PVL1 and PVL2 when the two slits are separated by quite a large distance. Here, we set *R*_1_ = 1900μm, *R*_2_ = *R*_1_ + *n λ*_SP_/2 (n = 1, 3, 5…) and investigated the corresponding field intensity patterns. The normalized results are depicted in Figure S8(a-h). Observably, due to the increased distance between PVL1 and PVL2, the innermost bright ring corresponding to *l*_1_ = 6 gradually evolved into a pentagon shape. This transformation can be interpreted as the larger distance making the inner PVL1 the relatively dominant contribution which is similar to the superposition of two OAM states with *l*_0_ = 1 and *l*_1_ = 6. The central deuterogenic vortex mode became more recognizable due to the more obvious amplitude difference between PVL1 and PVL2.


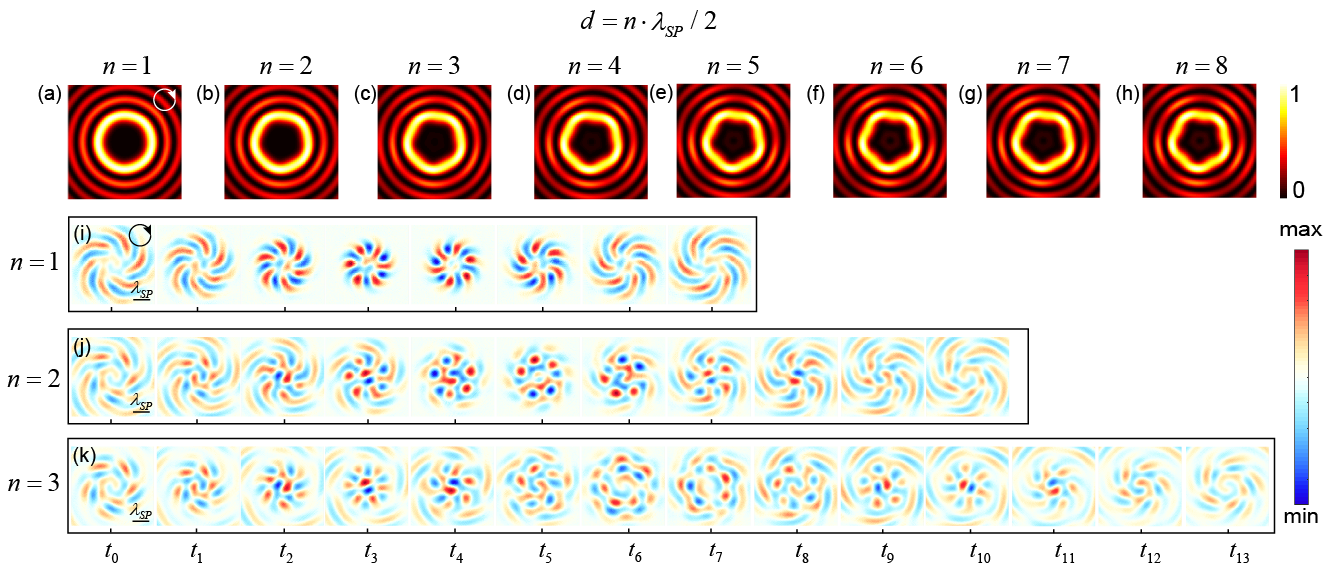


Figure S8. (a-h) Intensity distribution of the SP fields with PVL1 and PVL2 separated by different distance. (i-k) Temporal evolution of the excited plasmonic vortices with *n* =1, 2 ,3, respectively.

However, in terms of temporal revolution, the increased distance diminishes the temporal coherence between PVL1 and PVL2. A shown in Figure S8(i-k), the excited SPs are more distinctly separated in the time domain and corresponds to a longer evolution process as *n* increase. When , from *t*_0_ to *t*_5_, it is evident that the initial SP excitations from the inner PVL1 give rise to 12 converging spiraling wavefront threads surrounding the central two threads, signifying the formation of two vortices, *l*_1_ = 6 and *l*_0_ = 1. Subsequently, from *t*_6_ to *t*_9_, they develop into an outward-propagating spiraling wavefront and interfere with the SPs excited from the outer PVL2. From *t*_10_ to *t*_13_, the SPs field excited from PVL1 decayed and the excitations from PVL2 became the dominant contribution, undergoing the revolution and decay stages. Despite the increasing recognizability of the central deuterogenic vortex mode when the two slits are separated by quite a large distance (*n* = 6, 7, 8…), these structures lose the feasibility and generality for both basic researches and practical applications.

**Section 7: Generated plasmonic vortices in slit-pair-based PVL under different incidences.**

In order to manifest that the existence of the temporally deuterogenic vortex is a general strategy rather than a particular case, we also investigate the generated vortices of the same PVL with slit-pair separated by *λ*_SP_/2 under different incidences. Figure S8 illustrates the numerical and experimental results under LCP (*σ* = −1) incidence, from which we can see that there are also two lobes at the center besides the 12 converging spiraling wavefront threads (Figure S9c and S9f), but they can not be recognized directly from the field intensity distribution due to the destructive interference. From the phase patterns of Figure S8b and S8e, we can see the topological charges of the generated two plasmonics turns to *l*_0_ = −1 and *l*_1_ = −6. This is also embodied in the handedness of the wavefront flips in the temporal evolution snapshots compared with that of RCP incidence.


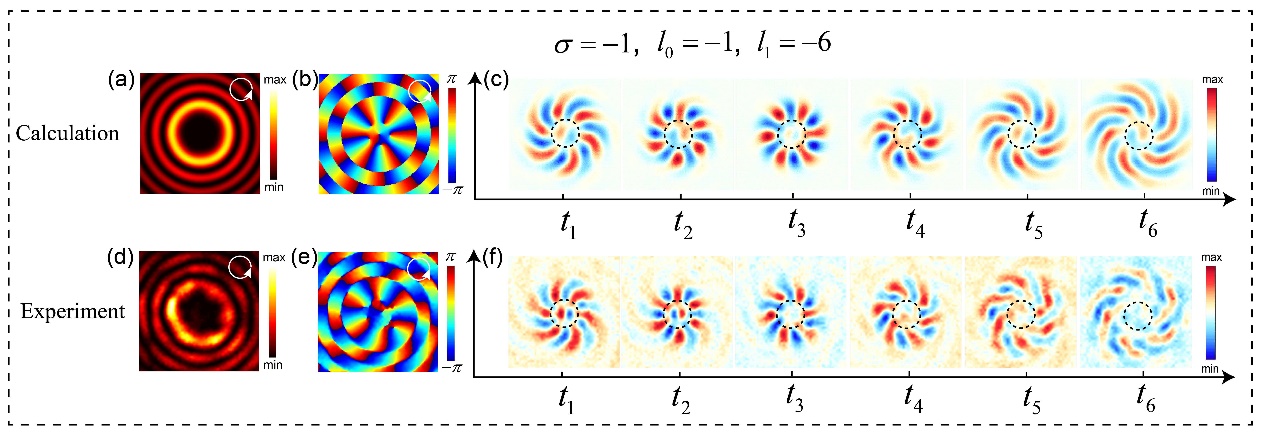


**Figure S9.** Generated plasmonic vortices under LCP incidence. (a–c) Numerical results of the SP fields intensity distribution (a), phase patterns (b) and the snapshots of the normalized amplitude field distribution in the *xy*-plane (c). (d–f) Experimental results.

In addition, when the incident beam has a topological charge *l*_in_, the temporally deuterogenic vortex can be also manipulated since the plasmonic OAM is converted from both the SAM and the OAM of incident vortex beams. As demonstrated by Figure S10, for the same PVL with geometric parameters (*g*, *α*_0_) = (7, π/2), we can get different compound vortices by changing the topological charges of incident optical vortex beams with the relation *l*_1_ = *σ*(*g* − 1)+ *l*_in_ and *l*_0_ = *σ*+ *l*_in_.


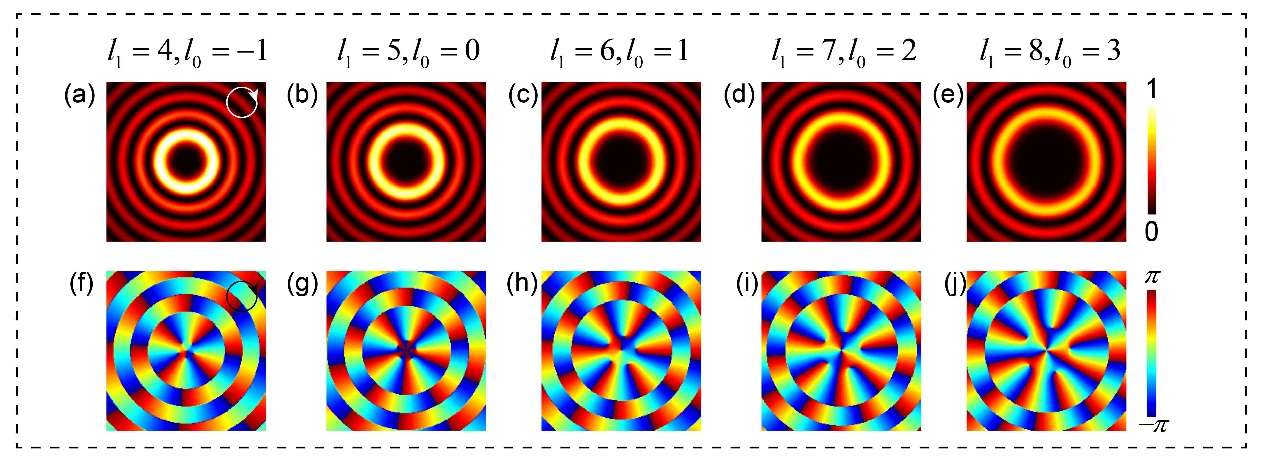


**Figure S10.** Generated plasmonic vortices under RCP vortex beam incidence. (a–e) Intensity distribution of the SP fields and (f–j) the corresponding phase profile of the generated PVs for the same PVL but different topological charge *l*_in_ of incident optical beams. (a) *l*_in_ = −2; (b) *l*_in_ = −1; (c) *l*_in_ = 0; (d) *l*_in_ = 1; (e) *l*_in_ = 2. *l*_1_ = *σ*(*g* − 1) + *l*_in_ and *l*_0_ = *σ* + *l*_in_ denote the two modes of generated PVs.
